# Endoplasmic Reticulum Unfolded Protein Response, Aging and Exercise: An Update

**DOI:** 10.3389/fphys.2018.01744

**Published:** 2018-12-05

**Authors:** Brisamar Estébanez, José A. de Paz, María J. Cuevas, Javier González-Gallego

**Affiliations:** ^1^Institute of Biomedicine (IBIOMED), University of León, León, Spain; ^2^Centro de Investigación Biomédica en Red de Enfermedades Hepáticas y Digestivas (CIBERehd), Madrid, Spain

**Keywords:** aging, elderly, endoplasmic reticulum stress, exercise, training, unfolded protein response

## Abstract

The endoplasmic reticulum (ER) is a dynamic and multifunctional organelle responsible for protein biosynthesis, folding, assembly and modifications. Loss of protein folding regulation, which leads to unfolded or misfolded proteins accumulation inside the ER lumen, drives ER stress (ERS) and unfolded protein response (UPR) activation. During aging, there is a decline in the ability of the cell to handle protein folding, accumulation and aggregation, and the function of UPR is compromised. There is a progressive failure of the chaperoning systems and a decline in many of its components, so that the UPR activation cannot rescue the ERS. Physical activity has been proposed as a powerful tool against aged-related diseases, which are linked to ERS. Interventional studies have demonstrated that regular exercise is able to decrease oxidative stress and inflammation and reverse mitochondrial and ER dysfunctions. Exercise-induced metabolic stress could activate the UPR since muscle contraction is directly involved in its activation, mediating exercise-induced adaptation responses. In fact, regular moderate-intensity exercise-induced ERS acts as a protective mechanism against current and future stressors. However, biological responses vary according to exercise intensity and therefore induce different degrees of ERS and UPR activation. This article reviews the effects of aging and exercise on ERS and UPR, also analyzing possible changes induced by different types of exercise in elderly subjects.

## Introduction

The endoplasmic reticulum (ER) plays an essential role in controlling various intracellular physiological functions, including protein translocation, protein folding, calcium homeostasis, and lipid biosynthesis (Naidoo, 2009a). Physiological conditions increase the protein folding demand, which may trigger loss of its regulation and leads to unfolded or misfolded proteins accumulation inside the ER lumen (Pereira et al., 2016). This accumulation drives ER stress (ERS) and unfolded protein response (UPR) activation. The UPR plays a main role in cell protection from stress and contributes to the reestablishment of cellular homeostasis; however, prolonged UPR activation could promote cell death (Fernández et al., 2015).

During aging, there is a decline in cell capacity to handle protein folding, accumulation, and aggregation, which may be, in part, due to a progressive failure of the chaperoning systems. Moreover, it seems that UPR activation cannot rescue the ERS, since some researches show a decline in many of UPR components (Naidoo, 2009b).

Physical activity has been proposed as a safe and effective therapeutic intervention in the elderly (Ogborn et al., 2014). It has been demonstrated that exercise attenuates oxidative stress and calcium imbalance (Bozi et al., 2016), as well as inflammation-related pathways, immune response or apoptotic cell death, and promotes autophagy (Rodriguez-Miguelez et al., 2014, 2015; Mejías-Peña et al., 2016, 2017). Numerous studies have also reported that exercise seems to improve some aging and ERS-related pathologies such as diabetes, neurodegenerative disease, sarcopenia, or cardiovascular alterations (Hong et al., 2017). Thus, physical training may be a potential strategy to reestablish ER homoeostasis in the elderly.

The aims of this review are to briefly identify the main effects of both aging and exercise on the ERS and UPR, and to specifically analyze changes on ERS and UPR following the performance of different types of exercise by elderly subjects.

## UPR Activation

**Figure 1:**
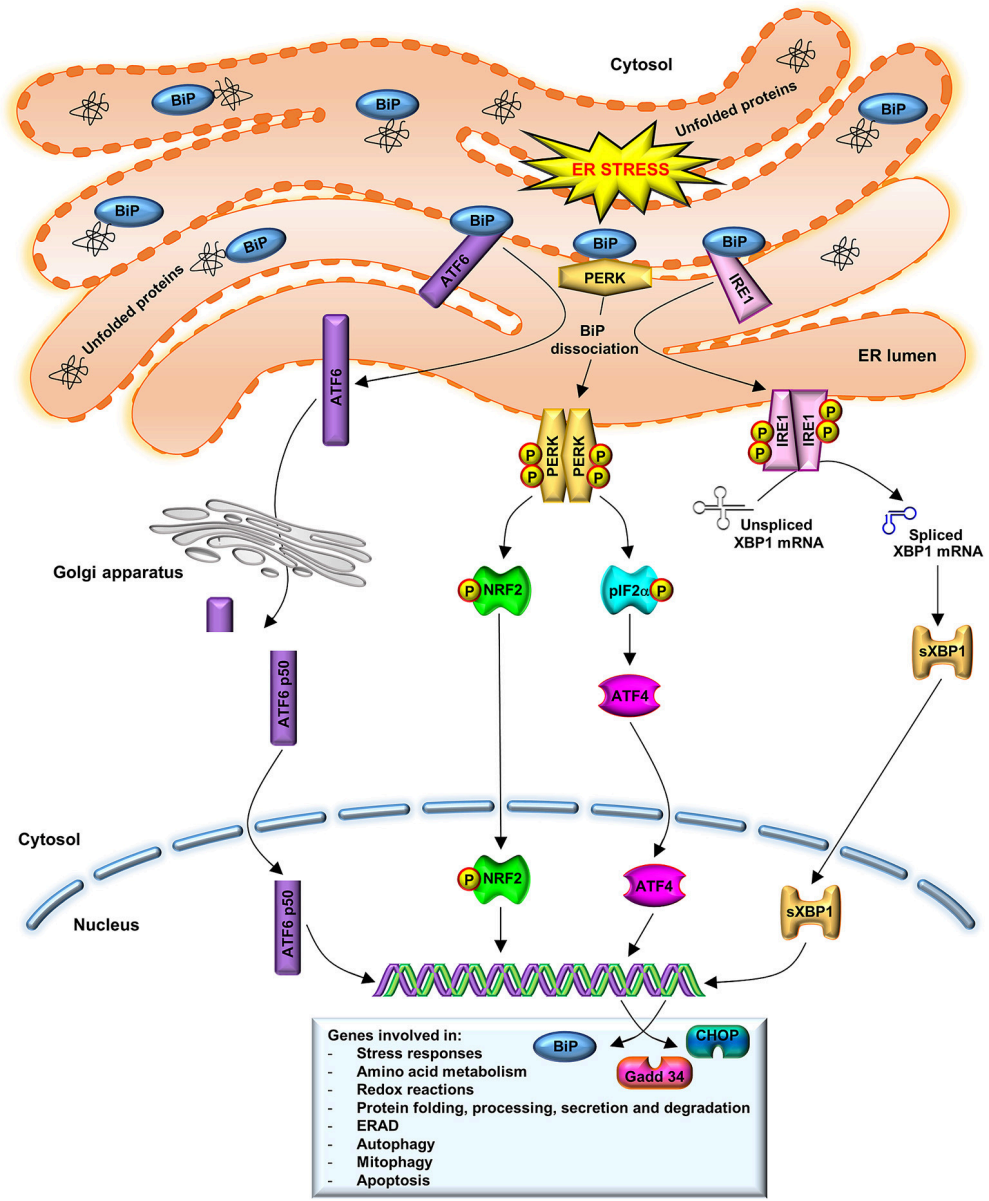
UPR activation. BiP dissociation from PERK, IRE1, and ATF6 initiates UPR through both PERK and IRE1 oligomerization and activation via trans-autophosphorylation and ATF6 translocation to the Golgi complex. Activated PERK phosphorylates eIF2a and stimulates ATF4 activity, which induces ERS target genes that are involved in amino acid metabolism, redox reactions, and protein secretion, thus to promote cell survival. A prolonged ATF4 activation leads to induction of CHOP. IRE1 dimerization catalyzes the splicing of XBP1 mRNA to synthetize a 54 kDa protein (sXBP1) which induces the expression of chaperones, as BiP, and components of the ERAD pathway. BiP also participates in cellular process such as autophagy, mitophagy, and apoptosis. In order to restore normal ER function, new synthesized BiP binds to PERK, IRE1, and to unfolded proteins, to refold them. ATF6 translocates to the Golgi, where it is cleaved in an active N-terminal 50 kDa domain. This active fragment is translocated to the nucleus where upregulates ER-associated chaperones and protein degradation factors, as well as CHOP and XBP1 expression.

The UPR has a cellular protective function. In order to decrease the ER protein load and, thus, the ERS, UPR drives to the upregulation ER-chaperones, such as binding immunoglobulin protein (BiP), to promote protein refolding. Moreover, UPR leads to the downregulation of protein translation through the activation of stress sensors such as protein kinase R-like ER kinase (PERK), inositol-requiring enzyme (IRE)1 and activating transcription factor (ATF)6. Additionally, UPR promotes the ER-associated degradation (ERAD) (Naidoo, 2009a). As illustrated in Figure 1, under physiological conditions, the luminal domains of BiP repress the activity of the three main stress sensors, PERK, IRE1, and ATF6, binding them (Crespo et al., 2012). However, ER lumen-accumulated unfolded proteins dissociate BiP from these effectors, which control the expression of downstream transcription factors: X-box binding protein (XBP)1 and ATF4 (Huang et al., 2015). These factors modulate the expression of chaperones or proteins involved in redox homeostasis, protein secretion or cell death programs (Senft and Ronai, 2015).

The first indicator of UPR is PERK activation, through its dimerization and autophosphorylation (Brown et al., 2014). PERK activated phosphorylates the eukaryotic translation initiation factor 2 subunit α (eIF2α), downregulating protein synthesis. ATF4, a PERK downstream protein, induces ERS target genes that are involved in the amino acid metabolism, redox reactions and protein secretion, to promote cell survival. Prolonged ATF4 expression leads to induction of transcription factor C/EBP homologous protein (CHOP) and further downstream growth arrest and DNA damage-inducible gene (Gadd)34, which forms a complex with protein phosphatase 1 to dephosphorylate eIF2α and recover the translation (Nakka et al., 2016). IRE1 dissociated from BiP leads to its dimerization and its endo-ribonuclease activity activation, catalyzing the non-canonical splicing of XBP1 mRNA to synthetize a 54 kDa protein (sXBP1), that induces BiP expression. In order to restore normal ER function, new synthesized BiP binds to PERK, IRE1 and unfolded proteins, to refold them (Nakka et al., 2016). Under stress conditions, ATF6 is translocated to Golgi, where it is cleaved in an N-terminal 50 kDa domain (ATF6-p50). This active fragment goes to the nucleus to upregulate ER-associated chaperones and protein degradation factors, as well as CHOP and XBP1 expression, protecting cells from chronic ERS (Fernández et al., 2015).

## UPR in Aging

In physiological conditions, the UPR increases both ER protein folding and degradation capacities to ensure quality control of the proteins assembled in the ER and reestablish ER homeostasis (Bozi et al., 2016). During aging, the function of UPR is compromised, since there is a progressive failure of the chaperoning systems and a decline in UPR components (see Table 1), so that UPR activation cannot rescue the ERS (Naidoo, 2009a,b).

**Table 1:** Effects of aging and exercise on UPR proteins.

**References**	**UPR proteins**		
	**(↑)**	**(↓)**	**(= )**
**UPR AND AGING**			
Baehr et al. (2016)			BiP^S, TA^; CHOP^S, TA^
Brown et al. (2014)	peIF2α^F^	eIF2α^F^	mRNA: sXBP1^F^; uXBP1^F^
Chalil et al. (2015a)	mRNA: ATF4^Os^; CHOP^Os^; sXBP1^Os^	
Chalil et al. (2015b)	BiP^EDL^; CHOP^G^; IRE1^G, EDL^; peIF2α^S, EDL^ mRNA: ATF4^G^; sXBP1^G^; uXBP1^G^	BiP^TA^; mRNA: ATF4^TA^; sXBP1^TA^; sXBP1/uXBP1^TA^	BiP^G, S^; CHOP^TA^; IRE1^TA, S^; peIF2α^TA, G^ mRNA: sXBP1/uXBP1^TA, G^
Drummond et al. (2011)			ATF4^VL^; peIF2α^VL^
Erickson et al. (2006)		BiP^L^
Gavilán et al. (2006)	mRNA: CHOP^Hp^	BiP^Hp^; mRNA: BiP^Hp^; PERK^Hp^; sXBP1^Hp^	mRNA: IRE1^Hp^
Ghosh et al. (2015)	ATF6^AT^; BiP^AT^; CHOP^AT^; pIRE1^AT^ mRNA: BiP^AT^; CHOP^AT^		mRNA: ATF4^AT^
Hussain and Ramaiah (2007)	CHOP^B, H, K, L, Lg, S^; Gadd34^B, Lg, L, K, H, Sp^; PERK^B, H, K, L, Lg, S^	ATF4^B, H, K, L, Lg, S;^ BiP^B, H, K, L, Lg, S^; eIF2α^B, Lg, L, K, H, Sp^; peIF2α^B, Lg, L, K, H, Sp^
Ikeyama et al. (2003)	CHOP^L^	
Jiao et al. (2012)			mRNA: ATF6^H^; BiP^H^; IRE1^H^; PERK^H^; uXBP1^H^
Jozsi et al. (2001)	mRNA: ATF4^VL^	
Li and Holbrook (2004)			CHOP^L^; peIF2α^L^ mRNA: CHOP^L^
Mihailidou et al. (2017)		BiP^P^; CHOP^P^
Naidoo et al. (2008)	CHOP^B^; Gadd34^B^	BiP^B^; peIF2α^B^; pPERK^B^
Naidoo et al. (2011)	CHOP^B^; Gadd34^B^; pPERK^B^		BiP^B^
Naidoo et al. (2014)	CHOP^P^; pPERK^P^		BiP^P^
O'Leary et al. (2013)	CHOP^TA^; mRNA: uXBP1^TA^		BiP^TA^
Ogborn et al. (2014)			mRNA: ATF6^VL^; BiP^VL^; Gadd34^VL^; IRE1^VL^; peIF2α^VL^; PERK^VL^; sXBP1^VL^; uXBP1^VL^
Rabek et al. (2003)		BiP^L^
Sreedhar et al. (2016)	BiP^H^; CHOP^H^	
Takeda et al. (2013)	ATF6^K^ cleaved; BiP^K^		CHOP^K^; pPERK^K^; sXBP1^K^
Tamura et al. (2017)	CHOP^G, P^; IRE1^G, P^		CHOP^S^; IRE1^S^
Torres-González et al. (2012)			mRNA: BiP^Lg^; sXBP1/uXBP1^Lg^
Wu et al. (2010)	peIF2α^K^ mRNA: CHOP^K^	mRNA: pPERK^K^	mRNA: BiP^K^
**UPR AND EXERCISE**			
Bozi et al. (2016)		BiP^H^	CHOP^H^
Cai et al. (2016)		pIRE1/IRE1^Hp(Ob)^	BiP^Hp(C, Ob)^; pIRE1/IRE1^Hp(C)^; pPERK/PERK^Hp(C, Ob)^
Chapados and Lavoie (2010)	mRNA: BiP^L(HFD)^		ATF6^L(SD, HFD)^; IRE1^L(SD, HFD)^; PERK^L(SD, HFD)^; uXBP1^L(SD, HFD)^; mRNA: BiP^L(SD)^; eIF2α^L(SD, HFD)^
da Luz et al. (2011)		mRNA: peIF2a^HFD(AT, L)^	pPERK^AT, L^ mRNA: peIF2a^SD(AT, L)^
Deldicque et al. (2013)	BiP^TA^; PERK^L, S^		BiP^L, S, P^; IRE1^S, TA, L, P^; PERK^P, TA^ mRNA: ATF4^TA, L^; CHOP^TA, L^; sXBP1^TA, L^; uXBP1^TA, L^
Hulmi et al. (2016)			BiP^G^; eIF2α^G^; IRE1^G^; peIF2α^G^; PERK^G^; sXBP1^G^ mRNA: eIF2α^G^; Gadd34^G^; pEIF2α^G^
Kang (2015)		BiP^Hp^; CHOP^Hp^
Khadir et al. (2016)		ATF6^AT^; BiP^AT, PBMCs, Pl^; peIF2α^AT, PBMCs^; pIRE1^AT^ mRNA: BiP^PBMCs^;	eIF2α^AT, PBMCs^; IRE1^AT^
Kim et al. (2010)	peIF2α^Hp(HFD{HR})^; pPERK^Hp(HR{LFD, HFD})^; sXBP1^Hp(LFD{LR}, *HFD*{*HR*})^ mRNA: ATF6^Hy(LFD{LR, HR}, *HFD*{*LR, HR*});^^Hp(HR{LFD, HFD});*Cx*(*LFD*{*LR*});*L*(*HFD*{*HR*})^; BiP^Hy(LFD{LR, HR}, *HFD*{*LR, HR*});*Hp*(*LFD*{*HR*}^, ^HFD{LR, HR})^; eIF2α^Hy(HR{LFD, HFD})^;^Hp(LFD{HR});*Cx*(*HR*{*LFD, HFD*});*L*(*HR*{*LFD, HFD*})^; XBP1^Hy(HFD{LR, HR});*Hp*(*HR*{*LFD, HFD*})^		ATF6 ^Hp(LFD{LR, HR}), *HFD*({*LR, HR*})^; BiP^Hp(LFD{LR, HR}, *HFD*{*LR*−*HR*})^; eIF2α^Hp(LFD{LR, HR}, *HFD*{*LR, HR*})^; peIF2α^Hp(LFD{LR, HR}, *HFD*{*LR*})^; PERK^Hp(LFD{LR, HR}, *HFD*{*LR*−*HR*})^; pPERK^Hp(LR{LFD, HFD})^; sXBP1^Hp(HR{LFD, HFD})^; uXBP1^Hp(LFD{LR, HR}, *HFD*{*LR*−*HR*})^ mRNA: ATF6^Hp(HR{LFD, HFD});*Cx*(*LFD*{*HR*})^;^L(HR{LFD, HFD})^; BiP^Cx(LFD{LR, HR}, *HFD*{*LR, HR*})^;^L(LFD{LR, HR}^,^HFD{LR, HR});*Hp*(*LFD*{*LR*})^; eIF2α^Hy(LR{LFD, HFD})^;^Hp(LFD{LR}, *HFD*{*LR, HR*});*Cx*(*LR*{*LFD, HFD*})^;^L(LR{LFD, HFD})^; XBP1^Hy(LFD{LR, HR});*Hp*(*LR*{*LFD, HFD*})^;^Cx(LFD{LR, HR}, *HFD*{*LR, HR*});*L*(*LFD*{*LR, HR*}, *HFD*{*LR, HR*})^
Kim et al. (2014)		BiP^G(HIT)^; mRNA: ATF4^G(HIT)^; CHOP^G(HIT)^	BiP^G(LIT)^; CHOP^G(LIT, HIT)^; PERK^G(LIT, HIT)^ mRNA: ATF4^G(LIT)^; CHOP^G(LIT)^
Memme et al. (2016)	CHOP^TA(1d, 2d, 3d, 5d, 7d)^ mRNA: ATF4^TA(2d, 3d)^; BiP^TA(1d, 2d, 3d, 7d)^; CHOP^TA(1d, 2d, 3d, 7d)^; sXBP1^TA(2d)^		BiP^TA(1d, 2d, 3d, 5d, 7d)^ mRNA: ATF4^TA(1d, 5d, 7d)^; BiP^TA(5d)^; CHOP^TA(5d)^; sXBP1^TA(1d, 3d, 5d, 7d)^
Pereira et al. (2016)	ATF6^EDL(OTRd)^;BiP^EDL(OTRd);S(OTR, OTRu)^; peIF2α/eIF2α^EDL(OTRd), S(OTR, OTRd;OTRu)^; pIRE1^EDL(OTRd);S(OTRd, OTRu)^; pPERK^EDL(OTRd, OTRu);S(OTR, OTRd;OTRu)^	ATF6^S(OTR, OTRd)^; BiP^EDL(OTRu);S(OTRd)^	ATF6^EDL(OTR, OTRu);S(OTRu)^; BiP^EDL(OTR);S(OTRd)^; peIF2α^EDL(OTR, OTRu)^; pIRE1^EDL(OTR, OTRu);S(OTR)^; pPERK^EDL(OTR)^
Pinto et al. (2017)	ATF6^Hy(OTRd)^; BiP^Hy(OTR, OTRd, OTRu)^; peIF2α^Hp(OTR/d)^; pIRE1^Hy(OTR, OTRu, OTRu)^; pPERK^Hy(OTR, OTRd, OTRu)^		ATF6^Hy(OTR, OTRu)^; peIF2α^Hy(OTRu, OTR)^
**UPR IN EXERCISE AND AGING**			
Baehr et al. (2016)	BiP^S(14d), TA(3d);^ CHOP^S, TA(3d−14d)^	
Drummond et al. (2008)			peIF2α^VL^
Drummond et al. (2011)	peIF2α^VL^		ATF4^VL^; peIF2α^VL^
Jozsi et al. (2001)	mRNA: ATF4^VL^	
Kang et al. (2013)		ATF6^Hp^; BiP^Hp^; CHOP^Hp^; peIF2α^Hp^; pPERK/PERK^Hp^; sXBP1^Hp^
Ogborn et al. (2014)	BiP^VL^; IRE1^VL^; PERK^VL^ mRNA: ATF6^VL^; eIF2α^VL^; IRE1^VL^; sXBP1^VL^	pPERK^VL^	peIF2α^VL^ mRNA: ATF4^VL^; BiP^VL^; CHOP^VL^; Gadd34^VL^ PERK^VL^; uXBP1^VL^
Um et al. (2008)	BiP^B^	

*AT, adipose tissue; B, brain; C, control; EDL, extensor digitorum longus; d, day; F, flays; G, gastrocnemius; H, heart; HFD, high fat diet; HIT, high intensity training; Hp, hippocampus; HR, high runners; Hy, hypothalamus; K, kidney; L, liver; LFD, low fat diet; Lg, Lung; LIT, low intensity training; LR, low runners; Ob, obese; Os, osteocytes; OTR, overtraining running; OTRd, downhill; OTRu, uphill; P, pancreas; PBMCs, peripheral blood mononuclear cells; Pl, plasma; S, soleus; SD, standard diet; Sp, spleen; TA, tibialis anterioris; VL, vasto lateralis*.

Several studies have shown decreased BiP levels in different tissues from aged mice and rats (Rabek et al., 2003; Erickson et al., 2006; Gavilán et al., 2006; Hussain and Ramaiah, 2007; Naidoo et al., 2008; Mihailidou et al., 2017). Other researches have indicated that BiP levels are not compromised in aged flies (Brown et al., 2014), several tissues from mice (Wu et al., 2010; Naidoo et al., 2011; Jiao et al., 2012; Torres-González et al., 2012) or rats (O'Leary et al., 2013; Baehr et al., 2016), and human muscle (Ogborn et al., 2014). However, other studies have revealed an increased BiP gene expression in mice heart and kidney (Takeda et al., 2013; Sreedhar et al., 2016) and a higher expression of both, BiP protein and mRNA, in mice adipose tissue (AT) (Ghosh et al., 2015). These discrepant results could be due to the evaluated tissues. In fact, Chalil et al. (2015b) after studying different types of muscles from aged mice found decreased BiP levels in tibialis anterioris (TA), increased in extensor digitorum longus (EDL) and remained without changes in gastrocnemius and soleus muscles.

With respect to PERK/eIF2α/ATF4/CHOP/Gadd34 pathway, again, different and contradictory results have been reported. Thus, PERK gene expresion was found to decrease in aged rats brain (Gavilán et al., 2006) and mice kidney (Wu et al., 2010), increase in mice pancreas (Naidoo et al., 2014), as well as in rat tissues (Hussain and Ramaiah, 2007), and remain unchanged in mice kidney and heart (Jiao et al., 2012; Takeda et al., 2013), as well as in human muscle (Ogborn et al., 2014). Even using the same tissue, such as brain, in aged mice both, decreases and increases, have been detected (Naidoo et al., 2008, 2011). eIF2α expression was diminished in flies (Brown et al., 2014), in rats tissues (Hussain and Ramaiah, 2007) and in mice cerebral cortex (Naidoo et al., 2008), and peIF2α increased in flies (Brown et al., 2014), rat liver (Li and Holbrook, 2004) and cells from renal cortex from aged mice (Naidoo et al., 2014). Moreover, although it has been indicated that eIF2α phosphorylation prevents oxidative stress-induced premature senescence (Rajesh et al., 2013), no changes were reported in aged human muscle (Drummond et al., 2008; Ogborn et al., 2014). In line with other UPR markers, peIF2α increased in soleus and EDL but did not change in TA and gastrocnemius muscles (Chalil et al., 2015b). Similar data have been found for ATF4. Thus, Hussain and Ramaiah (2007) showed decreased ATF4 levels in aged rat tissues, but other authors reported no changes in mice (Ghosh et al., 2015) or increases in mice osteocytes (Chalil et al., 2015a). In aged human muscles both, no changes or increases, have been reported (Jozsi et al., 2001; Drummond et al., 2011). Studies aimed to CHOP have demonstrated that this protein seems to be elevated in most of mice and rats aged tissues (Ikeyama et al., 2003; Gavilán et al., 2006; Hussain and Ramaiah, 2007; Naidoo et al., 2008, 2011, 2014; Wu et al., 2010; O'Leary et al., 2013; Chalil et al., 2015a,b; Ghosh et al., 2015; Sreedhar et al., 2016; Tamura et al., 2017). Nevertheless, some researches have shown no changes in this protein in mice kidney and rat liver (Li and Holbrook, 2004; Takeda et al., 2013), and even others have found both results depending on mice and rats muscles analyzed (Chalil et al., 2015b; Baehr et al., 2016; Tamura et al., 2017). Only in mice pancreas, Mihailidou et al. (2017) reported decreased CHOP levels. To our knowledge, no study has investigated CHOP state in elderly human subjects. Finally, Gadd34 increased in aged mice cerebral cortex (Naidoo et al., 2008, 2011) and in rat tissues (Hussain and Ramaiah, 2007); however, Gadd34 mRNA did not change in human muscles (Ogborn et al., 2014).

Concerning to the IRE1/XBP1 pathway, Ghosh et al. (2015) found an increase in pIRE1 in aged mice AT, while other authors described no changes in IRE1 mRNA expression in human muscle, rats brain and mice heart, respectively (Gavilán et al., 2006; Jiao et al., 2012; Ogborn et al., 2014). However, Chalil et al. (2015b) and Tamura et al. (2017) reported different results in IRE1 expression depending on mice muscle, with increases in gastrocnemius, EDL and plantaris and no change in TA and soleus. Similar differences were observed in XBP1 expression, where the unspliced and/or spliced forms increased in mice osteocytes, AT and kidney (O'Leary et al., 2013; Takeda et al., 2013; Chalil et al., 2015a), they were not modified in flies, human muscle, and mice lung and heart (Jiao et al., 2012; Torres-González et al., 2012; Brown et al., 2014; Ogborn et al., 2014) or experienced both, an increase and no changes, in mice muscle (Chalil et al., 2015b). Only in rat hippocampus a decrease in sXBP1 mRNA was found (Gavilán et al., 2006).

Finally, few studies have analyzed ATF6 expression in aged tissues. ATF6 was found to increase in mice kidney (Takeda et al., 2013) and AT (Ghosh et al., 2015) whereas no changes were reported in mice heart (Jiao et al., 2012) and human muscle (Ogborn et al., 2014).

## Exercise and UPR

Interventional studies have demonstrated that regular exercise is able to reverse ER dysfunctions (Passos et al., 2015). The UPR-energy availability link indicates that the UPR can mediate exercise-induced adaptation responses (Smiles et al., 2016) and it has been reported that muscle contraction is directly involved in UPR activation (Wu et al., 2011).

The stimulation of BiP mRNA or protein expression with exercise could depend on the training protocols, animal model, tissues analyzed or health status. Thus, 6-weeks endurance training upregulated BiP protein expression in mice muscle (Deldicque et al., 2013) and BiP mRNA expression in liver from rats fed with a high fat diet (HFD), but not in those rats fed with a standard diet (SD) (Chapados and Lavoie, 2010). Similarly, BiP mRNA levels increased with 7-days chronic contractile activity (CCA) (Memme et al., 2016) and after 3-weeks running, in mice hypothalamus and hippocampus (Kim et al., 2010). However, 8-weeks treadmill decreased BiP expression in rat heart (Bozi et al., 2016) and in mice hippocampus (Kang, 2015). In this line, a downregulation of BiP was also observed in peripheral blood mononuclear cells (PBMCs), plasma and AT from diabetic and non-diabetic humans after 12-weeks aerobic and resistance training (Khadir et al., 2016). Moreover, 5-weeks treadmill decreased BiP levels in muscle from rats subjected to training at high intensity (HIT), but not in muscle from rats trained at low intensity training (LIT) (Kim et al., 2014). Other researches did not observe changes in BiP expression. Thus, 8-weeks aerobic exercise did not modify BiP in control and obese rats hippocampus (Cai et al., 2016) and neither 6-weeks treadmill in mice soleus, liver, and pancreas (Deldicque et al., 2013).

In relation to UPR activation, some studies have demonstrated that several weeks of treadmill were not able to induce the PERK/eIF2α/ATF4/CHOP, IRE1/XBP1, or ATF6 pathways (Chapados and Lavoie, 2010; Deldicque et al., 2013; Kim et al., 2014; Hulmi et al., 2016). These results were independent of the tissue analyzed or the intervention used e.g., SD vs. HFD (Chapados and Lavoie, 2010; Deldicque et al., 2013) or LIT vs. HIT (Kim et al., 2014). However, other different results have also been reported. Thus, 6-weeks endurance training increased PERK levels in mice soleus and liver (Deldicque et al., 2013) and 3-weeks running increased ATF6 mRNA expression in mice brain (Kim et al., 2010). Finally, 8-weeks running in downhill, uphill, or without inclination modified pPERK, pIRE1, and ATF6 levels in mice hypothalamus and muscles (Pereira et al., 2016; Pinto et al., 2017). However, contradictory results were also found in the two previous studies, depending on the tissue and the type of protocol employed.

Several discrepancies were observed in eIF2α, ATF4, and CHOP gene expression. Thus, 12-weeks aerobic and resistance training increased peIF2α in human AT and PBMCs without concomitant changes in eIF2α gen expression (Khadir et al., 2016). In a study based in 3-weeks voluntary running with mice fed low-fat diet (LFD) or HFD, eIF2α mRNA expression was upregulated in brain and liver of high runners with both LFD and HFD, while peIF2α only increased in hippocampus of HFD-high runners (Kim et al., 2010). Similarly, 8-weeks running with different inclinations demonstrated that the downhill protocol increased peIF2α levels in mice EDL (Pereira et al., 2016) and hypothalamus (Pinto et al., 2017). By the contrary, 8-weeks swimming decreased peIF2α in HFD rat liver and AT, but not in control rats (da Luz et al., 2011). Different results were also observed in ATF4 mRNA. This marker remained unaltered in mice muscle after 6-weeks endurance training (Deldicque et al., 2013) or 5-weeks LIT-treadmill in rat muscle (Kim et al., 2014). However, although 5-weeks HIT-treadmill decreased ATF4 mRNA (Kim et al., 2014), the abundance of ATF4 mRNA increased in rat skeletal muscle at 2nd and 3rd days of CCA (Memme et al., 2016). In the same line, CHOP gene expression was upregulated almost the 7-days of CCA in rat muscle (Memme et al., 2016). Contrarily, 8-weeks treadmill downregulated CHOP levels in stress resistant mice hippocampus (Kang, 2015). Moreover, although 5-weeks HIT-treadmill also decreased CHOP mRNA levels in rat muscle, no protocol (LIT/HIT) was able to modify CHOP protein expression (Kim et al., 2014).

In regard to IRE1/XBP1 pathway, pIRE1/IRE1 ratio was lower in obese rats hippocampus after 8-weeks aerobic training (Cai et al., 2016). Furthermore, 12-weeks aerobic and resistance training decreased pIRE1 levels in human AT (Khadir et al., 2016). Likewise, Kim et al. (2010) showed that 3-weeks running increased uXBP1 mRNA expression in hypothalamus and hippocampus of both LFD- or HFD-low and high runners, but sXBP1 was only higher in hippocampus from LFD-low and HFD-high runners.

## Effects of Exercise on UPR in Aging

Physical activity has been proposed as a powerful and effective non-pharmacological approach for preventing and alleviating aged-related diseases, which are linked to ERS. Thus, it has been demonstrated that regular exercise has beneficial effects in the treatment of neurodegenerative diseases associated with misfolded proteins (Kang et al., 2013; Meijering et al., 2015). In fact, chronic exercise attenuates prolonged stress-induced hippocampal ERS and memory deficit (Kang, 2015).

A single resistance-exercise bout increased BiP protein expression in aged human muscle at 48 h, but BiP mRNA did not show changes at any time point (Ogborn et al., 2014). In the former research, authors observed an ATF6 mRNA upregulation at 24 and 48 h after the exercise (Ogborn et al., 2014). In an experiment carried out in aged rat, muscles underwent 7-days hindlimb unloading followed by reloading, BiP levels increased at the 3rd day of reloading in TA and at the 14th day of reloading on soleus muscles (Baehr et al., 2016). Otherwise, when effects of chronic training were studied, BiP levels increased in aged mice brain after 16-weeks treadmill (Um et al., 2008). However, 12-weeks treadmill downregulated BiP and ATF6 expression in presenilin (PS)2 mutant mice hippocampus (Kang et al., 2013).

With respect to PERK/eIF2a/ATF4/CHOP/Gadd34 pathway, Kang et al. (2013) found that 12-weeks treadmill decreased pPERK/PERK ratio in mutant PS2 mice hippocampus. However, a single bout of resistance exercise increased PERK levels, decreased pPERK and did not modify PERK and ATF4 mRNA in aged human muscle (Ogborn et al., 2014). Similar protocols of acute exercise either did not induce changes (Drummond et al., 2011) or increased ATF4 protein expression in muscle from old subjects (Jozsi et al., 2001). Concerning eIF2α, while Drummond et al. (2011) showed an increase in protein levels, other authors indicated no changes in eIF2α expression in aged human muscles after a single resistance-exercise bout (Drummond et al., 2008; Ogborn et al., 2014) or 12-week treadmill in PS2 mutant mice (Kang et al., 2013). Nevertheless, a single unaccustomed resistance-exercise bout upregulated eIF2α mRNA expression but had not effect on CHOP and Gadd34 mRNA levels in aged human muscle (Ogborn et al., 2014). Besides, 12-weeks treadmill decreased CHOP levels in mutant PS2 mice hippocampus (Kang et al., 2013) and, contrarily, the unloading/reloading protocol increased CHOP levels in aged rat soleus and TA (Baehr et al., 2016).

Regarding IRE1/XBP1 UPR pathway, Ogborn et al. (2014) reported increased IRE1 protein and mRNA levels, at 24 h, after a single resistance-exercise bout in aged human muscle. However, this study did not find an increase in uXBP1, but did it in sXBP1 mRNA levels (Ogborn et al., 2014). Conversely, Kang et al. (2013) found a decrease sXBP1 levels in mutant PS2 mice hippocampus after 12-weeks treadmill.

## Conclusions

ER, an essential organelle for cell homeostasis, plays a central role in cell death and survival signaling. In this review, we found discrepant results for each of the situations analyzed. Most of these conflicting findings may be due to differences in factors such as the use of young, adult or aged animal models, as well as the animal species used or their health status, the tissues analyzed or the acute and chronic training protocols carried out. Although the beneficial effect of exercise on UPR would depend on the modality and duration of exercise, results by different authors suggest that regular physical activity alleviates ERS both in middle-aged and old subjects. In this sense, it is possible that the response to exercise, both acute and chronic, can activate UPR signaling in order to alleviate ERS and decrease many of the UPR components in the elderly. However, we consider that further research is necessary to establish a direct relationship between UPR activation, exercise and aging, as well as to identify factors that may be influencing each of the conditions in which the UPR has been evaluated.

## Author Contributions

JG-G and MJC conceived and designed the manuscript. All authors contributed to the writing.

### Conflict of Interest Statement

The authors declare that the research was conducted in the absence of any commercial or financial relationships that could be construed as a potential conflict of interest.

## Abbreviations

AT: adipose tissue
ATF: activating transcription factor
BiP: binding immunoglobulin protein
B: brain
C: control
CHOP: C/EBP homologous protein
d: day
EDL: extensor digitorum longus
eIF2α: eukaryotic translation initiation factor 2 subunit α
EIF2AK3: eIF2α kinase 3
ER: endoplasmic reticulum
ERAD: endoplasmic reticulum associated degradation
ERS: endoplasmic reticulum stress
F: flays
G: gastrocnemius
Gadd: growth arrest and DNA damage-inducible gene
Grp: Glucose-regulated protein
H: heart
HFD: high fat diet
HIT: high intensity training
Hp: hippocampus
HR: high runners
Hy: hypothalamus
IRE1: inositol-requiring enzyme 1
K: kidney
L: liver
LFD: low fat diet
Lg: lung
LIT: low intensity training
LR, low runners, Ob: obese
Os: osteocytes
OTR: overtraining running
OTR/d: downhill
OTR/u: uphill
P: pancreas
PBMCs: peripheral blood mononuclear cells
peIF2α: eIF2α phosphorylated
PERK: protein kinase R (PKR)-like endoplasmic reticulum kinase
pIRE: IRE1 phosphorylated
PP1: protein phosphatase 1
pPERK: PERK phosphorylated
PS2: presenilin 2 mutant
S: soleus
SD: standard diet
Sp: spleen
sXBP1: spliced XBP1
TA: tibialis anterioris
UPR: unfolded protein response
uXBP1: unspliced XBP1
VL: vasto lateralis
XBP1: X-box binding protein 1.
